# Application of Postoperative Remote Follow-Up of CIED Based on the 5G Cloud Technology Support Platform in Areas With Underdeveloped Medical Resources

**DOI:** 10.3389/fcvm.2022.894345

**Published:** 2022-06-17

**Authors:** Lin Tong, Yu Long, Shiqiang Xiong, Jin Li, Wenchao Huang, Hanxiong Liu, Lin Cai

**Affiliations:** Department of Cardiology, The Third People's Hospital of Chengdu, Affiliated Hospital of Southwest Jiaotong University, Chengdu, China

**Keywords:** cardiovascular implantable electronic devices, COVID-19, follow-up, remote programming, telemedicine, in-office evaluation, 5G communication

## Abstract

Postoperative follow-up is crucial for the clinical management of patients carrying cardiovascular implantable electronic devices (CIED). However, in a plethora of underdeveloped areas of China, due to limited medical resources and associated economic costs, geographical restrictions, the outbreak of the COVID-19 pandemic, and various other reasons, the medical system is unable to meet the ever-increasing demand for long-term clinical follow-up and telemedicine services. Based on these challenges, postoperative remote follow-up of CIED based on the 5G-cloud technology support platform (5G-CTP) may have the potential to optimize the allocation of medical resources and provide patients with high-quality CIED follow-up services locally. These unique characteristics of CIED follow-up utilizing 5G-CTP are qualified to protect the safety of the patients in terms of both clinical safety and cyber security. Furthermore, during the COVID-19 pandemic, remote follow-up of CIED significantly reduces the risk of viral exposure to patients and medical staff while having the potential to improve the current situation of CIED postoperative follow-up.

## Introduction

Cardiovascular implantable electronic devices (CIED) are widely used in clinics for the monitoring and treating of cardiac arrhythmias, providing cardiac resynchronization therapy, and preventing sudden cardiac death. With the wide application of CIED in clinical settings, the total annual number of permanent pacemaker implantation in China has exceeded 80,000 by 2018 (1). Based on recent estimates, nearly one million patients are in need of postoperative follow-up management each year (1), with a rapidly growing clinical demand in China.

Routine follow-up requires patients to visit hospitals and is performed in an in-office evaluation manner. However, due to the increasing challenges on the available source of follow-up clinics, geographical isolations, the patient's inadequate awareness of postoperative follow-up schedules, and the COVID-19 pandemic, only 42% of the patients successfully completed the required follow-up within 2–12 weeks after CIED implantation, and most patients could not complete the whole routine follow-up schedules (1). Currently, due to these challenges, expert consensus and guidelines recommend the use of remote monitoring (RM) instead, to reduce the need for a hospital-based follow-up (1–3). The usage of RM has demonstrated several advantages, allowing clinical staff to monitor the status and vitals of patients, the working status and parameter information of CIED equipment, and detect early any clinical conditions that require urgent care. Nevertheless, regardless of these advantages and the robustness of the system to monitor clinical symptoms, RM is unable to proceed with any emergency intervention, such as the reprogramming of CIED, and therefore, cannot entirely take place in in-office evaluations. Furthermore, due to its high cost, the economic challenges for patients, the lack of telemedicine reimbursement policies, a shortage of dedicated follow-up specialists, and increasing third-party monitoring fees, fewer than 10% of the patients with implanted pacemakers participate in RM in China. RM service was just introduced into China several years. Its expense has not been included in the payment scope of the basic medical insurance. Currently, only the United States and Germany have realized full reimbursement (1). Thus, routine in-office follow-up still remains the primary strategy for CIED management in China. As mentioned above, in a variety of geographically isolated regions, especially during the COVID-19 pandemic, the patient's access to in-office follow-up centers remains largely challenging. Therefore, a novel strategy for follow-up needs to be exploited to improve the management of CIED follow-up in such areas with underdeveloped medical resources.

## 5G-Cloud Technology Support Platform

**Figure 1:**
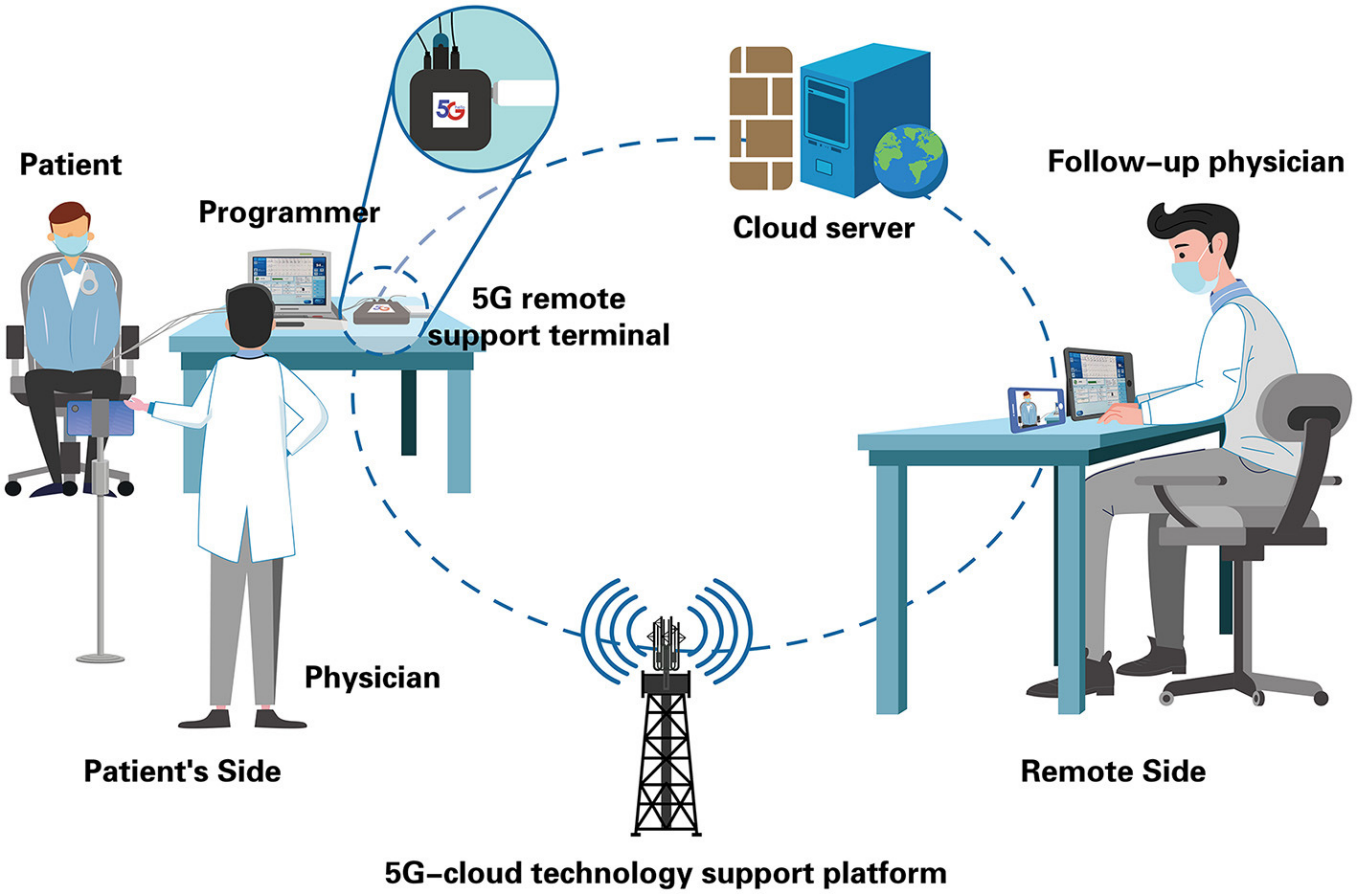
Schematic representation of real-time remote programming for cardiovascular implantable electronic devices (CIED) follow-up through the 5G cloud technology support platform (5G-CTP). The patient's side is equipped with a CIED programmer, a 5G remote support terminal, and a physician; the remote's side is equipped with a tablet installed with a cloud follow-up APP, as well as a follow-up physician (clinical device specialist). The physician initials the cloud follow-up by externally connecting the 5G remote support terminal to the programmer, and putting the wand to the patient's device. When confirming the communication is stable, the physician gets in contact with the follow-up physician and gives a briefing. Follow-up physician logs into the cloud follow-up APP to establish a remote connection with the 5G remote support terminal on the patient's side. Then he has complete control of the programmer, and is able to interrogate, test, and reprogram the device remotely as needed. The whole remote service system is deployed on the cloud server with multilayer protection, including firewalls, customized antivirus, and vulnerability scanning, along with intrusion detection.

Postoperative remote follow-up of CIED based on the 5G-cloud technology support platform (5G-CTP), is based on a cloud technology support platform, jointly developed by China Telecom. The communication in this system is supported by the fifth generation mobile communication technology (5G) network offered by China's big three telecom carriers (China Telecom, China Mobile, and China Unicom). The 5G-CTP can effectively establish a secure connection between the patient's side (programmer, 5G remote support terminal) and the remote side (*via* mobile phone or tablet installed with a cloud follow-up application, APP), while the whole remote service system is deployed on cloud servers (Figure 1). A dedicated follow-up specialist on the remote side logs into the cloud follow-up APP to conduct remote interrogation, parameter testing, and reprogramming appropriately. One of our previous studies has reported the clinical use of real-time remote programming in pacemakers based on 5G-CTP. The whole remote session was performed efficiently and safely without any adverse events (4).

## Composition of Cloud Follow-Up System

**Figure 2:**
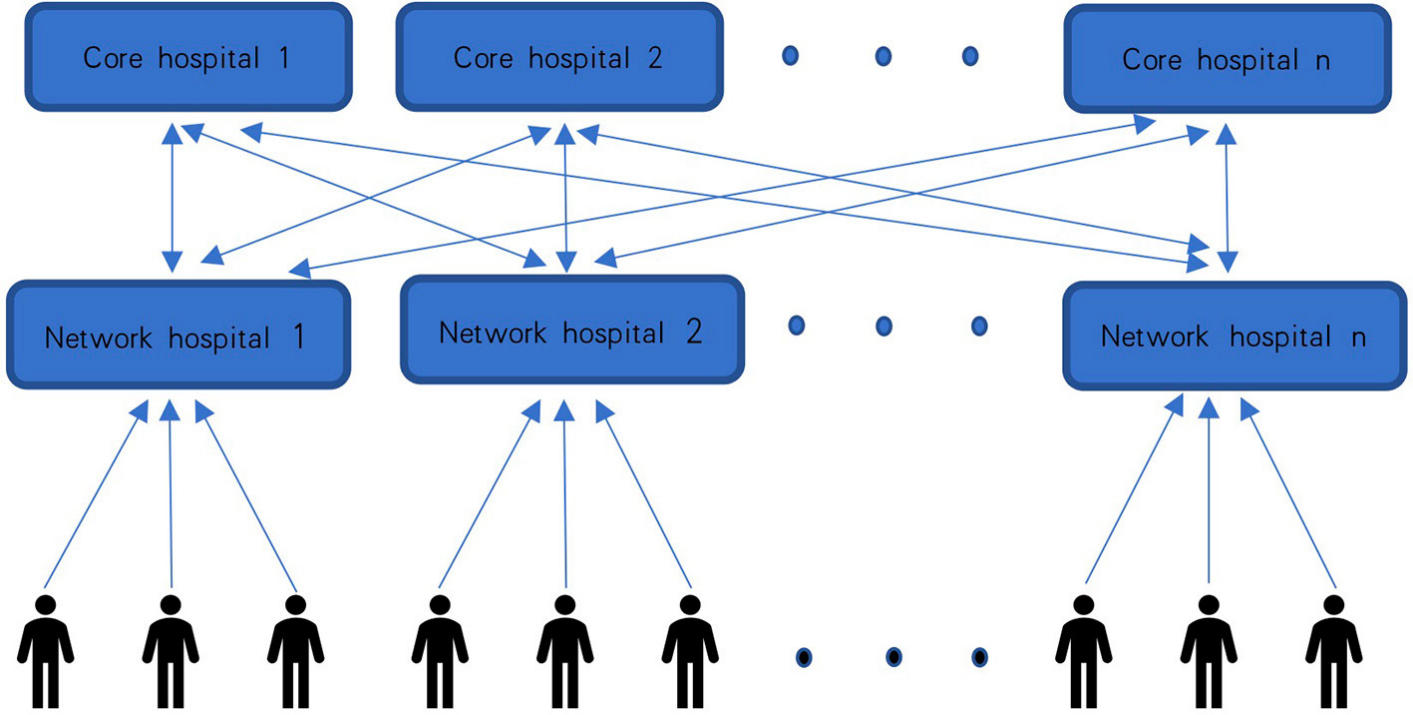
Schematic diagram of regional collaborative cloud follow-up system. Core hospitals (having follow-up clinics or device specialists) establish a regional collaborative cloud follow-up, one-to-one or one-to-many, system with the network hospitals (lack of follow-up clinics or device specialists). A regional collaborative cloud follow-up system covers the whole region and provides follow-up management for the local CIED patients.

Based on the wide availability of the 5G network in China, core hospitals that have follow-up clinics or device specialists can establish a regional collaborative cloud follow-up, one-to-one, or one-to-many system with the network hospitals (primary care institutions) that lack follows up clinics or device specialists, through the 5G-CTP (Figure 2). As for the equipment, the network hospital is equipped with a CIED programmer, 5G remote support terminal, ECG monitor, first-aid equipment, and a physician; the CIED cloud follow-up center of the core hospital is equipped with mobile electronic devices, such as mobile phones or tablets, installed with cloud follow-up APP, as well as follow-up specialists (clinical device specialists).

## Cloud Follow-Up Process

After obtaining the patients' written consent, the physician in the local network hospital initiates the cloud follow-up by externally connecting the 5G remote support terminal to the programmer and putting the wand on the patient's device. When confirming the communication is stable, the physician gets in contact with the remote device specialist in the core hospital and gives a briefing (Figure 1).

On the core hospital's side, after receiving the contact, the remote follow-up specialist logs into the cloud follow-up APP on a mobile electronic device *via* a two-step verification process: Step 1: log into the designated account, and Step 2: use a specific access password for the second verification to log into the cloud follow-up APP, to establish a remote connection with the 5G remote support terminal on the patient's side. Then the remote follow-up specialist has complete control of the programmer and is able to interrogate, test, and reprogram the device remotely as needed.

The whole process of cloud follow-up is saved *via* screen recording. The log documents containing the duration and details of remote operations allow users to audit the logs later.

## Protection of Patient Data Security During Cloud Follow-Up

To ensure effective patient safety protection, a dual password authentication login is utilized. First, the remote follow-up specialist logs into the designated account using a password and then uses the access password for the second verification to log into the cloud follow-up APP. The whole remote service system is deployed on the cloud servers with multilayer protection, including firewalls, customized antivirus, and vulnerability scanning, along with intrusion detection. To protect the security of data transferring, the system utilizes a 2048-bit RSA asymmetric key exchange and autonomous P2P data transfer protocol building on AES encryption protocols.

## Protection of Patient Medical Safety During Cloud Follow-Up

Before the cloud follow-up session, the physician on the patient's side informs the patient and his family of the function, benefits, and risks of the cloud follow-up. After obtaining the written consent form from the patient and his family, the local physician and the remote follow-up specialist are connected *via* telephone or video in real-time, while the patient can also communicate with the follow-up specialist to eliminate the patient's doubts and concerns.

Apart from the proper establishment of connections, the physician on the patient's side should be able to identify life-threatening changes in ECG, such as severe bradycardia (<40 beats/min), ventricular tachycardia, or ventricular fibrillation. In case of capture loss in a pacemaker-dependent patient, the onsite physician should immediately reprogram the device to emergency VVI pacing for bradycardia by activating the “VVI” key located on the programmer. In case of ventricular fibrillation, the onsite physician should immediately activate the “Shock” key located on the programmer. If the connection signal is delayed or interrupted, it will revert the device to the original settings.

## The Reliability and Feasibility of Cloud Follow-Up

In order to evaluate the technical reliability and feasibility of cloud follow-up in CIED patients, we have conducted a multicenter, observational trial (ChiCTR2100046883). Until now, 325 CIED patients from 13 hospitals in Sichuan Province of China have been enrolled. The preliminary results found that the compliance with routine in-office evaluation in this region was only 60.6%. None of the patients were engaged in RM. A total of 94 (31.9%) PPMs, 4 (25.0%) ICDs, and 5 (35.8%) CRTs were reprogrammed remotely as appropriate. All cloud follow-up sessions were performed efficiently and safely, with no adverse events. 89.8% of the patients preferred cloud follow-up for future device management (unpublished data). The Basic operational flow has been created and will be examined in future studies.

## Discussion

The CIED implantation procedure and postoperative follow-up are mainly performed in tertiary hospitals, while the majority of primary care institutions do not have the qualifications to perform CIED follow-up. The implementation of cloud follow-up based on 5G-CTP promotes tertiary hospitals' medical resources sinking into primary-level medical and health units, which enables primary care institutions to provide convenient follow-up services for the local CIED patients.

During the COVID-19 pandemic, the availability of CIED follow-up clinics and resources is dramatically reduced. To this extent, the Cloud follow-up system, which allows patients to complete follow-up in local primary care institutions, may reduce the risk of COVID-19 transmission and cross-infection during cross-regional transportation.

The common factors that contribute to noncompliance with the follow-up schedule primarily include the medical condition of the patient's residence, the distance to the follow-up clinic, and the restriction of population mobility during the COVID-19 pandemic. Table 1 summarizes the distance, time, transportation mode, and non-clinical expenses for CIED patients living in underdeveloped medical areas traveling to and from between core hospitals and their residences in Sichuan province. With the use of the cloud follow-up system, the network hospital owns the ability to provide routine in-office evaluation for the local CIED patients. Therefore, the cloud follow-up system has the potential to reduce the cross-regional flow of patients, shorten the transportation distance between patients' residences and the follow-up clinic, and reduce time and financial costs for patients. With all these characteristics, cloud follow-up might improve the follow-up management in patients living in areas with underdeveloped medical resources.

**Table 1:** The distance, time, transportation mode and non-clinical expenses of patients to and from core hospitals in areas with underdeveloped medical resources in Sichuan.

**Areas with underdeveloped medical**	**Round trip**	**Transportation**	**Round trip**	**Non-clinical expenses**
**resources**	**distance (km)**	**mode**	**time (hour)**	**(traffic+hotel+food) (yuan)**
Ganzi Tibetan Autonomous Prefecture	About 600	Plane	2–4	2,000–4,000
		Car + train	16–20	300–500
		Self-driving	7–10	300–500
Aba Tibetan Autonomous Prefecture	About 600	Plane	2–3	1,100–1,500
		Cars + trains	16–20	300–500
		Self-driving	about 10	about 500
Liangshan Yi Autonomous Prefecture	About 900	Plane	2–4	700–900
		Train	22–25	500
		Self-driving	13–15	500–800
Panzhihua City	About 1300	Plane	2–3	About 1,500
		Train	25–27	About 500
		Self-driving	18–20	1,000–1,200
Guangyuan City	About 600	Plane	—	—
		Train	about 9	About 200
		Self-driving	8–10	600–800
Bazhong City	About 700	Plane	2–3	About 600
		Train	About 9	About 300
		Self-driving	8–10	600–800
Dazhou City	About 800	Plane	2–3	600–900
		Train	about 8	150–200
		Self-driving	9–11	700–900

## Limitations of Cloud Follow-Up

Regardless of the advancements and advantages of this follow-up system, cloud follow-up did not reduce the frequency of in-office evaluation. CIED patients still need to finish in-office evaluations as guidelines recommended (5). Furthermore, on the one hand, although this technology enables patients to achieve local follow-up, on the other hand, also increases the workload of clinical device specialists in tertiary hospitals. Currently, the cloud follow-up of CIED is still under clinical study. More evidence-based studies and comprehensive experience in clinical practice are needed to further improve the operation process and protocols of cloud follow-up.

## Conclusion

Regular follow-up is crucial for patients carrying CIED. The compliance rate of routine follow-up is discouraging in a plethora of regions in China, especially during the era of the COVID-19 pandemic. The cloud follow-up model provides patients with high-quality CIED follow-up services locally while also providing continuous medical training for local physicians at the same time. Regardless of the limitations of the cloud follow-up, the system is characterized by its potential to improve the postoperative follow-up management and clinical prognosis in a certain population of CIED patients. It is worthwhile to coordinate multilateral efforts from the medical community to establish cloud follow-up systems in areas with limited medical resources.

## Data Availability Statement

The original contributions presented in the study are included in the article/supplementary material, further inquiries can be directed to the corresponding authors.

## Author Contributions

YL and SX were major contributors to the drafting of the manuscript. JL and WH put forward constructive comments and suggestions. LT, SX, and HL revised the manuscript for important intellectual content. LC designed the study and finally approved the manuscript submitted. All authors read and approved the final manuscript.

## Funding

This work was supported by the Science and Technology Department of Sichuan, China (Grant Numbers: 2021YJ0215 and 2020YJ0483), Chengdu High-level Key Clinical Specialty Construction Project, and the National Natural Science Foundation of China (31600942).

## Conflict of Interest

The authors declare that the research was conducted in the absence of any commercial or financial relationships that could be construed as a potential conflict of interest.

## Publisher's Note

All claims expressed in this article are solely those of the authors and do not necessarily represent those of their affiliated organizations, or those of the publisher, the editors and the reviewers. Any product that may be evaluated in this article, or claim that may be made by its manufacturer, is not guaranteed or endorsed by the publisher.
